# Autoantibodies in Japanese patients with ocular myasthenia gravis

**DOI:** 10.1002/mus.27103

**Published:** 2020-11-12

**Authors:** Akiko Nagaishi, Tomoko Narita, Mark Woodhall, Leslie Jacobson, Patrick Waters, Sarosh R. Irani, Angela Vincent, Hidenori Matsuo

**Affiliations:** ^1^ Department of Neurology Nagasaki Kawatana Medical Center Nagasaki Japan; ^2^ Nuffield Department of Clinical Neurosciences, Oxford Autoimmune Neurology Group University of Oxford Oxford UK; ^3^ Nuffield Department of Clinical Neurosciences University of Oxford Oxford UK

**Keywords:** anti‐acetylcholine receptor antibody, cell‐based assays, LRP4, MuSK, ocular myasthenia gravis

## Abstract

**Introduction:**

The majority of patients with myasthenia gravis (MG) initially present with ocular symptoms, but it is difficult to predict which cases will remain as ocular MG (OMG) or will progress to generalized MG. Herein we evaluated the serologic profile of Japanese OMG and its relationship with clinical features.

**Methods:**

Seventy‐three patients with OMG from five Japanese myasthenia gravis (MG) centers were enrolled. Live cell‐based assays (CBAs) were used to determine the presence of autoantibodies (Abs) to clustered adult (2α, β, δ, ε) and fetal (2α, β, δ, γ) acetylcholine receptor (AChR) isoforms, muscle‐specific receptor tyrosine kinase (MuSK), and lipoprotein receptor–related protein‐4 (LRP4).

**Results:**

Thirty‐four of 73 (46.5%) serum samples were positive for Abs against both the adult‐type and fetal‐type AChR, as expected, but 7 (9.6%) and 2 (2.7%) were positive only for fetal or adult AChR‐Abs, respectively. Four (5.4%) samples were positive for MuSK‐Abs, but two of these also contained antibodies to fetal AChR or LRP4. Twenty‐six (35.6%) samples were seronegative.

**Discussion:**

Abs against fetal‐specific AChR, MuSK, and LRP4 are found in some patients with OMG. Future studies attempting to predict conversion from ocular symptoms to generalized MG may benefit from measurement of these antibodies.

**Abbreviations**
Ab: antibody
AChR: acetylcholine receptor
CBA: cell‐based assay
cDNA: complementary DNA
DMEM: Dulbecco’s modified Eagle’s medium
EGFP: enhanced green fluorescent protein
GMG: generalized myathenia gravis
IgG: immunoglobulin G
IT: immunotherapy
LRP4: lipoprotein receptor–related protein‐4
MGFA: Myasthenia Gravis Foundation of America
MuSK: muscle‐specific receptor tyrosine kinase
OMG: ocular myathenia gravis
RIA: radioimmunoassay
RT: room temperature

## 1 INTRODUCTION

Patients with myasthenia gravis (MG) have varied clinical courses, but the majority initially present with ocular symptoms, and it is difficult to predict which of these cases will remain ocular MG (OMG) or progress to generalized MG (GMG). Acetylcholine receptor antibodies (AChR‐Abs) are found in 80% to 90% of GMG and usually around 50% of OMG cases^1^, ^2^; however, in one Japanese study (that included 22% OMG cases), 79.8% of MG patients had AChR‐Abs, whereas 12.9% of GMG and no OMG patients had muscle‐specific receptor tyrosine kinase (MuSK)‐Abs.^3^ Other reports have described MuSK‐Abs in individual OMG patients.^4^, ^5^, ^6^, ^7^

In human muscle, the AChR subunits largely switch from the fetal type (2α, β, δ, γ) to the adult type (2α, β, δ, ε) by birth. However, γ‐subunit expression persists into adulthood in the extraocular muscles^8^ and it is possible that OMG patients have antibodies to fetal as well as adult AChRs.

Recently, live cell‐based assays (CBAs) have been established to detect autoantibody binding to native clustered AChR, MuSK, and lipoprotein receptor–related protein‐4 (LRP4). These live CBAs offer better sensitivity than fixed CBAs or radioimmunoassays (RIAs),^9^, ^10^, ^11^ because they detect binding of divalent immunoglobulin G (IgG) antibodies to adjacent native antigens anchored on the cell surface and do not detect binding to irrelevant intracellular epitopes.^12^, ^13^, ^14^, ^15^ In this study, we used live CBAs to test MG‐associated antibodies, including adult and fetal AChR subtypes, in 73 Japanese OMG patients.

## 2 METHODS

We obtained approval from the ethics committee of Nagasaki Kawatana Medical Center (Nagasaki, Japan) and permission from the ethics committee of each institution that participated in this study. All subjects provided informed consent.

### 2.1 Patients

Consecutive MG patients at five member institutes for MG research in the Intractable/Rare Disease Control Division of the Japanese Ministry of Health, Labor and Welfare were enrolled in this study during January to December 2015. Diagnoses of OMG were based on patients' symptoms and results of an electrophysiologic exam of repetitive nerve stimulation and/or jitter measurements and an edrophonium test. Only patients with a diagnosis of OMG (grade “I” in the Myasthenia Gravis Foundation of America [MGFA] classification^16^) were considered eligible. Patients were excluded if their clinical course was shorter than 2 years or they had thymoma. Patients were included regardless of their immunotherapy (IT) status.

### 2.2 Live cell–based assays

Live CBAs^14^, ^15^ were performed in the neuroimmunology laboratory at Oxford University: hamster embronic kidney 293T cells were transiently transfected with plasmids using polyethylenimine. For the clustered AChR assay, cells were transfected with complementary DNAs (cDNAs) expressing 4 subunits of human AChR at an α:β:δ:ε/γ:rapsyn ratio of 2:1:1:1:1 with enhanced green fluorescent protein (EGFP)‐rapsyn to cluster the AChRs. For MuSK‐Ab detection, MuSK cDNA was tagged with EGFP. For the LRP4 assay, cDNAs expressing human LRP4 and a chaperone protein (low‐density lipoprotein receptor–related protein–associated protein‐1) were cotransfected at a 5:1 ratio.

Twenty‐four hours posttransfection, all cells were incubated with patient sera (diluted 1:20 with Dulbecco’s modified Eagle’s medium [Sigma‐Aldrich, St. Louis, Missouri] containing 20 mmol/L HEPES with 1% bovine serum albumin [BSA; Sigma‐Aldrich]) for 1 hour at room temperature (RT). After washing, the cells were fixed with 4% paraformaldehyde for 10 minutes at RT, rinsed twice, and incubated with a secondary goat anti‐human IgG Fc(γ) antibody (Thermo Fisher Scientific, Rockford, Illinois), diluted 1:750, for 45 minutes at RT. Next, a third antibody layer was applied (donkey anti‐goat IgG[H+L] Alexa Fluor 568; Life Technologies, Eugene, Oregon), diluted 1:750, for 45 minutes at RT. After the final washes, coverslips were mounted using fluorescence mounting medium (DakoCytomation, Cambridge, UK) containing 1% 4′,6′‐diamidino‐2‐phenylindole dichloride (nuclear stain) and examined using a fluorescent microscope.

The CBAs were scored as described by Leite et al^13^: 0 = no labeling; 1 = weak labeling of some transfected cells; 2 = moderate labeling of approximately 20% to 50% of transfected cells; 3 = moderate/strong labeling of approximately 50% to 80% of transfected cells; and 4 = strong labeling of almost all transfected cells.

### 2.3 Statistical analysis

Statistical analysis was performed using GraphPad Prism 8 version 8.2.1 (GraphPad, La Jolla, California). The Mann‐Whitney *U* test was used for analyses according to the age of onset. The significance of statistical differences between subgroups was evaluated using Fisher’s exact test. Differences were considered significant at *P* < .05.

## 3 RESULTS

Serum samples from 73 OMG patients were tested. Forty‐seven patients were seropositive for CBA antibodies to both adult and fetal AChR (n = 34), fetal AChR only (n = 7), adult AChR only (n = 2), or MuSK (n = 4) (Table 1). Two of the MuSK‐Ab–positive patients also had antibodies to fetal AChR or LRP4, respectively. The patients with positive antibodies had an older median age of onset than the seronegative group (57 vs 45; *P* = .0297), but there was no gender difference.

**TABLE 1:** Results of CBA assays in all patients

CBA diagnosis	N (%)	Female, n (%)	Onset age, median (range), years	Disease duration at sampling, median (range), years	Abnormal thymus on CT/MRI	Immunotherapy‐naive, n (%)	Asymptomatic at sampling, n (%)
Total	73 (100.0)	36 (49.3)	51 (2‐83)	8 (2‐52)	4	28 (38.4)	21 (28.8)
Seropositive	47 (64.4)	25 (53.2)	57 (2‐83)	7 (2‐46)	4	18 (38.3)	16 (34.0)
Adult+fetal AChR	34 (46.6)	21 (61.8)	60 (2‐83)	6.5 (2‐46)	4	14 (41.2)	11 (32.4)
Fetal AChR	7 (9.6)	2 (28.6)	52 (38‐67)	7 (4‐16)	0	1 (14.3)	2 (28.6)
Adult AChR	2 (2.7)	1 (50.0)	5, 41	5, 46	0	1 (50.0)	0 (0.0)
Fetal AChR and MuSK	1 (1.4)	0 (0.0)	63	5	0	1 (100.0)	1 (100.0)
MuSK	2 (2.7)	1 (50.0)	45, 57	12, 15	0	0 (0.0)	1 (50.0)
MuSK and LRP4	1 (1.4)	1 (100.0)	69	4	0	1 (100.0)	0 (0.0)
LRP4	0 (0.0)						
Triple seronegative	26 (35.6)	11 (42.3)	45 (4‐70)	8 (2‐52)	0	9 (34.6)	5 (19.2)

Abbreviations: AChR, acetylcholine receptor; CBA, cell‐based assay; CT, computed tomography; LRP4, lipoprotein receptor–related protein‐4; MRI, magnetic resonance imaging; MuSK, muscle‐specific tyrosine kinase.

**FIGURE 1:**
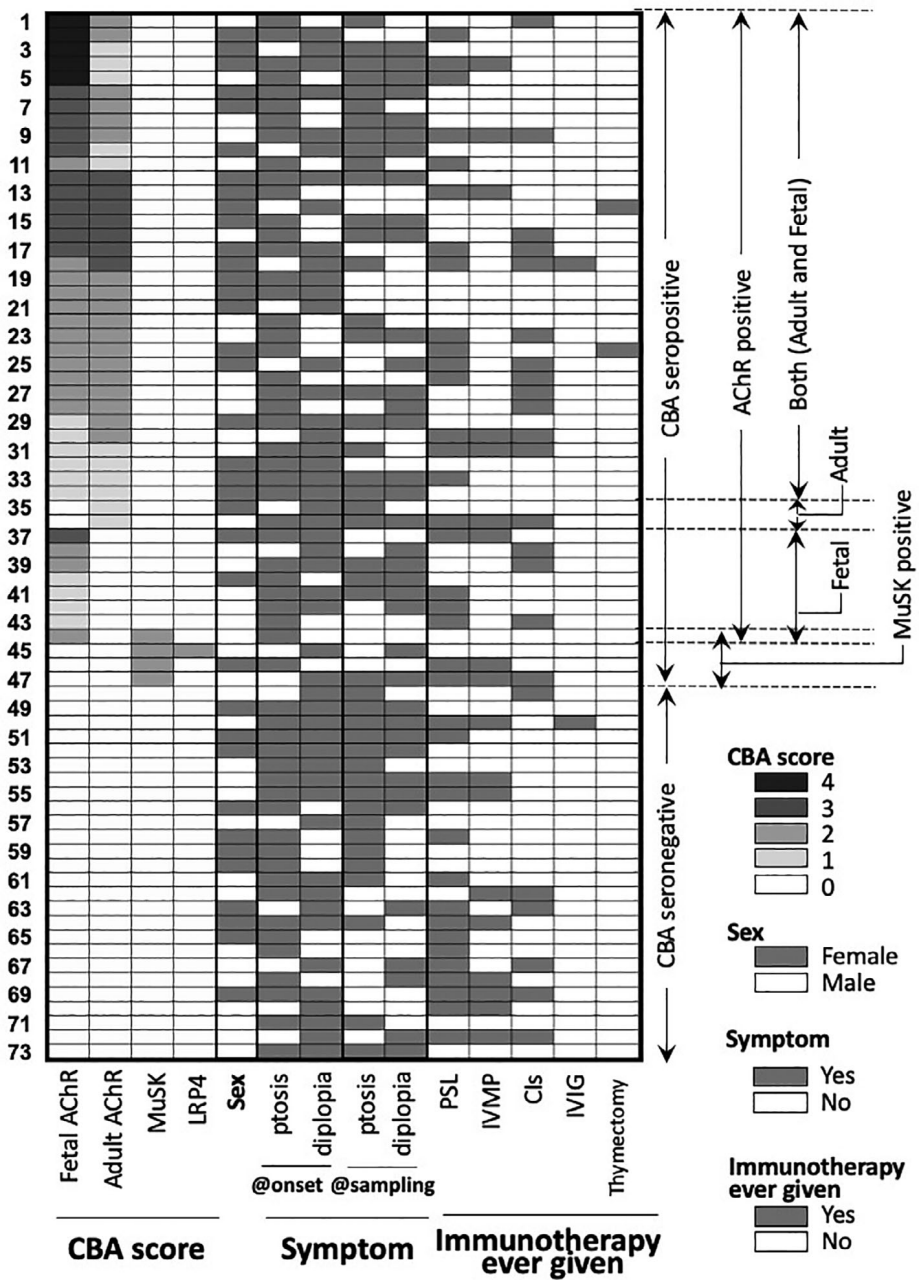
Heatmap of the relationships between CBA results and histories of patients with ocular myasthenia gravis. Most patients treated with CIs received tacrolimus, except for two patients who received cyclosporine A (nos. 23 and 34). Abbreviations: AChR, acetylcholine receptor; CBA, cell‐based assay; CI, calcineurin inhibitor; IVIg, intravenous immunoglobulin; IVMP, intravenous methylprednisolone; LRP4, lipoprotein receptor–related protein‐4; MuSK, muscle‐specific tyrosine kinase; PSL, oral prednisolone

The CBA results, symptoms, and immunotherapy histories of each patient are shown in Figure 1. Immunotherapy‐naive patients comprised 38.3% of the seropositive group and 34.6% of those who were seronegative. In comparison, only one of seven patients in the fetal AChR‐Ab–positive group was immunotherapy‐naive compared with 14 of 34 who had both adult and fetal AChR‐Abs, but this difference was not significant. Table 2 presents symptoms at onset and at sampling. Overall, there were few changes in clinical expression of the ocular symptoms (ptosis and diplopia); in six of the fetal AChR‐Ab–positive group patients, ptosis was present at onset compared with only three at sampling.

**TABLE 2:** Symptoms of patients at onset and at sampling

	At onset	At sampling
(1) Fetal AChR‐Ab alone	7	
Both ptosis and diplopia (%)	4 (42.9)	3 (42.9)
Ptosis alone (%)	2 (28.6)	0 (0.0)
Diplopia alone (%)	1 (14.3)	2 (28.6)
All ptosis (%)	6 (85.7)	3 (42.9)
All diplopia (%)	5 (71.4)	5 (71.4)
Asymptomatic (%)	—	2 (28.6)
(2) Any AChR‐Ab except for (1)	36	
Both ptosis and diplopia (%)	15 (41.7)	16 (44.4)
Ptosis alone (%)	13 (36.1)	7 (19.4)
Diplopia alone (%)	8 (22.2)	2 (5.6)
All ptosis (%)	28 (77.8)	23 (63.9)
All diplopia (%)	23 (63.9)	18 (50.0)
Asymptomatic (%)	—	11 (30.5)
(3) MuSK‐Ab^a^	4	
Both ptosis and diplopia (%)	0 (0.0)	1 (25.0)
Ptosis alone (%)	2 (50.0)	0 (0.0)
Diplopia alone (%)	2 (50.0)	1 (25.0)
All ptosis (%)	2 (50.0)	1 (25.0)
All diplopia (%)	2 (50.0)	2 (50.0)
Asymptomatic (%)	—	2 (50.0)
(4) Triple antibody‐negative	26	
Both ptosis and diplopia (%)	12 (46.2)	8 (30.7)
Ptosis alone (%)	7 (26.9)	9 (34.6)
Diplopia alone (%)	6 (23.1)	4 (15.3)
All ptosis (%)	20 (76.9)	17 (65.3)
All diplopia (%)	19 (73.0)	12 (46.1)
Asymptomatic (%)	—	5 (19.2)

Abbreviations: AChR, acetylcholine receptor; CBA, cell‐based assay; CT, computed tomography; LRP4, lipoprotein receptor–related protein‐4; MRI, magnetic resonance imaging; MuSK, muscle‐specific tyrosine kinase.

^a^ Includes 1 case double‐positive with fetal AChR and 1 case double‐positive with LRP4.

Finally, we compared results of these assays with those at diagnosis and at sampling using RIA for AChR‐ and MuSK‐Abs. All patients positive for AChR‐Abs on CBA had been positive for AChR‐Abs on RIA at onset, but three were no longer positive by RIA at time of CBA testing. In addition, two of the patients positive for MuSK‐Abs on CBA and 12 who had been designated positive for AChR‐Abs on RIA had had very low RIA titers (≤1.0 nmol/L) at onset and were negative at sampling.

## 4 DISCUSSION

We used CBAs to evaluate the serologic profiles of Japanese OMG patients, 61.6% of whom had undergone a variety of immunotherapies. As expected, 46.6% of the patients were positive for both clustered adult and fetal AChR‐Abs, but another 17.8% had antibodies only binding to fetal AChR, adult AChR, or MuSK (2 of the latter also with fetal AChR or LRP4 antibodies).

Most patients with MG have antibodies that bind effectively to solubilized mixtures of adult and fetal AChRs by RIA, but, increasingly, cell‐based assays are used to measure antibodies for increased sensitivity and specificity.^13^ These CBAs have the advantages that only extracellular epitopes are exposed to the antibodies, the antigen is expressed on the membrane in native confirmation, and high expression of the antigen allows antibodies to bind divalently to adjacent targets, as is highly relevant at synapses such as the neuromuscular junctions.^13^

It was surprising to find seven patients with only fetal AChR antibodies, although, in one previous study,^17^ fetal‐only AChR antibodies were seen in 12 of 200 (6%) OMG patients. The role of fetal AChR antibodies is poorly understood; unusually, the extraocular muscle fibers have both adult (epsilon subunit containing) and fetal (gamma subunit containing) AChRs, suggesting that the fetal AChR antibodies could be pathogenic, but expression of the epsilon subunit is much higher than the gamma subunit.^18^ Moreover, mothers with predominant fetal AChR antibodies do not have severe ocular symptoms and some are asymptomatic.^19^ Therefore, an alternative explanation is that relatively low levels of adult AChR‐Abs are present initially, but are adsorbed by the adult AChRs in the extraocular and levator muscles, leaving the fetal AChR antibodies in the circulation. In patients with higher levels of antibodies, typical of generalized MG, adsorption would be less likely to deplete adult AChR‐Abs.

MuSK‐Abs were first described in patients with severe generalized symptoms who often presented with bulbar and respiratory failure,^20^ but some present first with ocular symptoms only. In a recent study,^15^ MuSK‐MG patients who were only positive by CBA (ie, RIA‐negative) had mild symptoms, with MGFA grades 1 or 2 (5 of 13 OMG), compared with only 26.9% of RIA‐positive MuSK‐MG with grades over 2. It may be that testing for MuSK‐Abs by CBA in OMG patients without AChR‐Abs will prove helpful in selected cases.

This study has some limitations. Further examination of human ocular muscles and the relationships between specific antibodies and OMG is needed. In addition, we used sera collected years after the onset of disease, and many patients had already been given immunotherapies. Some patients may have undergone a change in the serologic profile after treatment or over the natural disease course. Twelve of the cohort had been positive previously for AChR antibodies on RIA, but had low titers (<1.0 nmol/L); such patients could have become antibody‐negative after immunotherapies, but caution is necessary with such low titers.

In conclusion, we have observed Abs against MuSK, LRP4, and fetal‐ and adult‐specific AChR‐Abs in OMG patients. Further studies attempting to predict conversion from ocular symptoms to generalized MG may benefit from the measurement of these antibodies.

## CONFLICT OF INTEREST

University of Oxford and A.V. hold a patent (2001) for MuSK antibody assays, licensed to Athena Diagnostics USA; A.V. has received a proportion of royalties for antibody tests.

## ETHICAL PUBLICATION STATEMENT

We confirm that we have read the Journal’s position on issues involved in ethical publication and affirm that this report is consistent with those guidelines.
